# Central African Field Epidemiology and Laboratory Training Program: building and strengthening regional workforce capacity in public health

**Published:** 2011-12-14

**Authors:** Gervais Ondobo Andze, Abel Namsenmo, Benoit Kebella Illunga, Ditu Kazambu, Dieula Delissaint, Christopher Kuaban, Francois-Xavier Mbopi-Kéou, Wilfred Gabsa, Leopold Mulumba, Jean Pierre Bangamingo, John Ngulefac, Melissa Dahlke, David Mukanga, Peter Nsubuga

**Affiliations:** 1 Ministry of Public Health, Cameroon; 2 Ministry of Public Health, Central African Republic; 3 Ministry of Health, Democratic Republic of Congo; 4 Central Africa FELTP, Cameroon; 5 University of Yaoundé, Faculty of Medicine and Biomedical Sciences, Cameroon; 6 Ministry of Higher Education, Cameroon; 7 Ministry of Agriculture, DRC; 8 Centers for Disease Control and Prevention, Atlanta, USA; 9 African Field Epidemiology Network, Kampala, Uganda

**Keywords:** epidemiology, education, training, public health, Central Africa

## Abstract

The Central African Field Epidemiology and Laboratory Training Program (CAFELTP) is a 2-year public health leadership capacity building training program. It was established in October 2010 to enhance capacity for applied epidemiology and public health laboratory services in three countries: Cameroon, Central African Republic, and the Democratic Republic of Congo. The aim of the program is to develop a trained public health workforce to assure that acute public health events are detected, investigated, and responded to quickly and effectively. The program consists of 25% didactic and 75% practical training (field based activities). Although the program is still in its infancy, the residents have already responded to six outbreak investigations in the region, evaluated 18 public health surveillance systems and public health programs, and completed 18 management projects. Through these various activities, information is shared to understand similarities and differences in the region leading to new and innovative approaches in public health. The program provides opportunities for regional and international networking in field epidemiology and laboratory activities, and is particularly beneficial for countries that may not have the immediate resources to host an individual country program. Several of the trainees from the first cohort already hold leadership positions within the ministries of health and national laboratories, and will return to their assignments better equipped to face the public health challenges in the region. They bring with them knowledge, practical training, and experiences gained through the program to shape the future of the public health landscape in their countries.

## Introduction

The Central African Field Epidemiology Training Program (CAFELTP) was established in October 2010 to enhance capacity for applied epidemiology and public health laboratory services in three countries: Cameroon (CAE), Central African Republic (CAR), and the Democratic Republic of Congo (DRC). The aim of the program is to develop a trained public health workforce to assure that acute public health events are detected, investigated, and responded to quickly and effectively. CAFELTP is part of the Bill and Melinda Gates Foundation-supported Surveillance in Central Africa (SURVAC) project, which was designed to improve disease surveillance and response with a specific focus on vaccine-preventable diseases in Central Africa.

The Central Africa region has a long history of notable disease outbreaks such as meningitis, yellow fever, measles, viral hemorrhagic fevers (Ebola and Marburg), and monkey pox[1–7]. For example, in the last decade outbreaks of meningitis occurred in Cameroon in areas of the Northwest (2001, 2004), North (2004), Southwest (2004), the Far North (2007, 2009) and Adamawa (2010) [4,8]. Other nearly eradicated diseases, such as poliomyelitis and guinea worm, have re-emerged in the region to have a significant public health impact [9,10]. HIV/AIDS and tuberculosis are also prevalent in the region [1–3]. Vectors responsible for deadly diseases such as malaria and trypanosomiasis are common in Central Africa [11,12].

Although classical Master of Public Health (MPH) programs are offered in DRC and Cameroon, the focus of these programs is not on field epidemiology and public health laboratory training and, at present, no formal MPH program is offered in CAR. The public health challenges in the region have fueled the need to build workforce capacity to address the unmet need for field epidemiologists and public health laboratory epidemiologists to perform outbreak investigations, surveillance, and research [13].

## Overview of CAFELTP

### History of CAFELTP

The CAFELTP is a sub-regional program established at the Faculty of Medicine and Biomedical Sciences, University of Yaoundé I, based in Yaoundé, Cameroun. The mission of the program is to strengthen the public health workforce capacity in Cameroun, Central African Republic and the Democratic Republic of Congo to respond to public health emergencies such as outbreaks, natural disasters, and emerging infectious diseases. Its vision is to strengthen public health surveillance and laboratory systems through 1) developing leadership in public health; 2) educating and training public health professionals in epidemiology and laboratory sciences; 3) supporting public health laboratory services in surveillance and field investigations; 4) supplying technical support and advisory services to key stakeholders such as the MOH; and 5) improving communications and networking within the country and throughout the region.

The program offers two tracks: Field Epidemiology and Laboratory Management. The CAFELTP began with 18 residents in the first cohort: five each from CAE and CAR, and eight from DRC. Of the 18 residents, 12 follow the field epidemiology track and six follow the laboratory track. Ten of the epidemiology residents are medical doctors and two are veterinarians. The six laboratorians are medical biologists. All didactic courses are conducted at the University of Yaoundé 1, in Cameroon. There are four didactic blocks which account for 25% of the 2-year long program. In between the didactic blocks the residents conduct field activities to learn while offering public health service in their home countries under supervision from the in country stakeholders and the under guidance of the Resident Advisors.

### Funding and Key Partnerships

CAFELTP is a partnership between the Bill and Melinda Gates Foundation (BMGF), the CDC Foundation, the World Health Organization (WHO), the Centers for Disease Control and Prevention (CDC), the African Field Epidemiology Network (AFENET) and three Central African countries (Cameroon, the Democratic Republic of Congo and the Central African Republic). Though the program is hosted at the University of Yaoundé I, it is integrated within the ministries of health of the three participating countries. CAFELTP is directed by the Director of the Department of Disease Control Unit at the Ministry of Public Health, Yaoundé, Cameroon. Furthermore, Country Directors from CAR and DRC are members of the steering committee and actively participate in the residents’ field-based activities by identifying suitable field sites and supervising the residents during their field placements.

The program is financially supported by the BMGF and the United States Agency for International Development (USAID). One of the BMGF areas of focus includes vaccine-preventable diseases, neglected tropical diseases, and other infectious diseases. The BMGF has committed to financially supporting 15 residents annually for a period of 5 years. USAID currently sponsors the three veterinary residents from DRC. The program works closely with AFENET and CDC Atlanta to receive technical oversight and administrative support.

### Description of the Components of the Program

CAFELTP is a 2-year public health capacity building training program using the FELTP “learn-by-doing” model. Upon completion of the program, students will receive a Masters of Science degree in Applied Epidemiology or Laboratory Management from the University of Yaoundé I. The program consists of 25% didactic and 75% practical (field based activities). The didactic portion of the program consists of lectures, conferences and seminars, tutorials, local, national and international colloquiums, workshops and congresses. It is organized into modules, with the first module consisting of the core public health courses in epidemiology, laboratory, surveillance, and biostatistics for the first 6 weeks.

After completion of these modules, the epidemiology residents are assigned to departments within the ministries of health and agriculture in their respective countries. The laboratory residents are assigned to a national laboratory or other accredited laboratory institution. The residents are supervised and mentored by a senior epidemiologist, a public health specialist, or a laboratory scientist. The residents are required to do four practical projects over the 2-year period. The duration of the field-based practicum varies from 3to 4months at a time. Over the 2-year period, the residents are expected to complete the following requirements: evaluation of a surveillance system, evaluation of a health program, evaluation of a health management project, outbreak investigation, field project, data surveillance analysis report, dissertation, submission of two publishable manuscripts, and presentations at national, regional or international conferences. Table 1 shows the distribution of field sites within the ministries of health. 

**Table 1:** Distribution of field sites within the 3 ministries of health

Country	Name of Sites	Number of Residents
Cameroon	Expanded Program on Immunization (EPI)	2
	Division of Disease Control	1
	National Laboratory	2
Central African Republic	Expanded Program on Immunization (EPI)	2
	Division of Disease Control and Prevention	1
	Bangui Community Hospital (Hôpital Communautaire de Bangui)	2
Democratic Republic of Congo	Expanded Program on Immunization (EPI)	1
	Veterinary Lab at the Ministry of Agriculture	2
	Division of Disease Control	3
	National Research Laboratory	2

## Achievements of CAFELTP

Although the program is still in its infancy, there are several achievements worth noting. During the program's short duration, CAFELTP residents have already participated in six outbreak investigations, including poliomyelitis and cholera in DRC and cholera in various regions of Cameroon. During outbreak investigations, the residents have assisted the Ministries of Health with data collection and analysis, and summaries of epidemiological information. The residents are also involved in weekly surveillance meetings within the departments of disease control in their respective countries. The nature of emerging and re-emerging infectious diseases in the Congo Basin and Central African region makes it likely that the students will have many opportunities to participate and become more proficient in outbreak investigation and response.

The Central Africa Program became the first FELTP to provide the “Strengthening Laboratory Management Toward Accreditation” (SLMTA) training to the laboratory residents in July 2011. This training was conducted in collaboration with the CDC International Laboratory Branch. The laboratory residents received training on managing and improving laboratories and laboratory workforce to meet accreditation standards according to the WHO guidelines. The residents conducted baseline assessments at their assigned laboratories, and identified an improvement project to help the laboratory become accredited in the future. This capacity-building activity provides the residents with the opportunity to work hand-in-hand with the SLMTA trainers and the laboratory directors and managers toward laboratory accreditation.

### Contributions of CAFELTP to Building Long-Term Sustainability in Public Health

Like the similar West African Field Epidemiology and Laboratory Training Program (WAFELTP), the CAFELTP is addressing public health challenges using a regional approach. In addition to responding to outbreak investigations, the residents have evaluated 18 public health surveillance systems and public health programs, and completed 18 management projects. These projects include evaluations of public health surveillance systems for measles, maternal and neonatal tetanus, acute flaccid paralysis, and bacterial meningitis. Through these various activities, information is being shared to understand similarities and differences in the region, which can lead to new and innovative approaches for public health response. The program provides opportunities for regional and international networking in field epidemiology and laboratory practice, particularly for countries that may not currently have the resources to host their own program.

Several of the residents from the first cohort already hold leadership positions within the ministry of health and national laboratory in their respective countries, and will return to their assignments better equipped to face the public health challenges in the region. They bring with them knowledge, training and experiences gained to help shape the future of the public health landscape in their countries. The program also promotes long-term epidemiological and laboratory capacity building when trainees will return to the program as field supervisors, trainers, and mentors to future cohorts, passing on their knowledge to the next generation of public health leaders, and therefore expanding the pool of highly qualified public health professionals in the region. The cross border nature of the program will enhance core capacities for implementation of the revised International Health Regulations in this highly epidemic prone region; the residents can collaborate on outbreaks that cross the borders of the three countries [14,15].

### Sustainability of CAFELTP

Similar to the other more established programs, CAFELTP must confront the inevitable challenges of continued funding and long-term sustainability. A critical issue for developing a long-term sustainability plan is maintaining and continuing the program services after the end of funding. The initial funding from the BMGF is expected to last 5 years from the onset of the program. Ensuring that relevant career opportunities are available for graduates is another important issue. The literature shows that a lack of post-graduate training and opportunities for career advancement, low salaries and conditions of services are some of the factors for migration of healthcare workers [9,10]. However, greater financial incentives, opportunities for professional and personal growth are some of the factors for recruitment and retention of healthcare workers.

CAFELTP team along with its partners will work on a career portfolio, which would allow for promotion and salary increase based on candidate qualifications and experience upon graduation from the program. Opportunities in government for highly qualified field epidemiologists and public health laboratorians should be made available.

To be sustainable in the long term, the government entities and institutions in the countries must assume ownership of the program with multi-sector support (i.e., from the ministries of health, ministries of agriculture, ministries of higher education and other institutions). Involvement of the governments in sponsoring residents is a step forward to ensure sustainability of the program. However, the institutions also need to be ready and capable of providing the necessary resources, the administrative environment, and long-term dedication to make field epidemiology and laboratory management training work.

The CAFELTP management team is also working with the CDC country offices in the region and other public health organizations to provide technical and financial support to the program. To ensure that workforce capacity remains in the region, residents are required to sign a contract committing to work in their country's government service for a minimum number of years after graduation (typically 3 to 5 depending on each country's policy).

Strategies for sustainability of the Central Africa program include lower costs, increased efficiencies, and increasing the visibility of the program by marketing the CAFELTP and its results to various stakeholders in the region. For example CAFELTP can endeavor to participate in projects that have a high public health impact, or consider combining all didactics into one session at the beginning to reduce travel costs, or even requiring students to pay some portion of their tuition fees. However, the program trains individuals in one of the most resource-challenged regions of the world and therefore self-funding may not be a reality in the near term. Other options could be to recruit part-time students who are already employed, and who will be able to assume their tuition fees.

The program is working on finding options from multiple sources at various levels. One of these options could be a combination of new funding for categorical programs to combat priority diseases, including the US President′s Emergency Plan for AIDS Relief (PEPFAR), the Global Fund to Fight Tuberculosis, AIDS and Malaria, and the Global Alliance for Vaccines and Immunizations (GAVI) Alliance. These programs might contribute to strengthening human capacity by supporting initiatives such as training programs in field epidemiology. Thus, the training programs can meet and sustain their goals with an added positive effect on countries′ abilities to improve health in many areas [16].

## Challenges

Since its inception in October 2010, the program has been running well despite experiencing some challenges. The scarcity of applied epidemiology and other public health textbooks available in French makes it difficult to provide reference and support materials to the residents. The limited resources available from governments and other donors creates difficulty in addressing critical public health needs and limits the program's ability to recruit and train a critical mass of CAFELTP graduates in the three countries. As a result, at this time it is not possible to ensure an adequate number of well-trained public health staff for deployment when events of public health concern occur. However, there are ongoing efforts to explore strategies to recruit more representatives from the various provinces of each country into CAFELTP.

## Conclusion

In light of the demonstrated public health challenges within the region, the training model of the FELTP will continue to be developed in Central Africa. The limited available resources and integrated nature of health issues has lead to the regional strategy currently being implemented. Contributing factors such as underdeveloped infrastructure, limited access to quality health care, limited laboratory capacity, as well as social and environmental influences indicate that some solutions can only be realized in the long term. There is a need to improve the capacity for applied epidemiology and laboratory management at all levels of government. The program is directly meeting the acute need for public health specialists, which will lead to strengthening public health workforce capacity to prevent diseases in the region.

As the program becomes more established, strong government and university support will be needed to achieve its objectives. Forums for current residents and alumni to showcase their work will raise awareness of the value of FELTPs to governments and institutions in the region. Public health issues can be addressed by these competently-trained public-health professionals, increase the country′s ability to combat AIDS, tuberculosis, and malaria as well as diarrheal diseases, maternal mortality, and chronic diseases, and to address the International Health Regulations and other disease priorities of the ministries of health [6].

As countries meet the challenge of institutionalizing their programs, the CAFELTP concept may increasingly be recognized as a model for sustainable regional public health capacity development and expansion of FELTPs into other countries in the Central Africa region. The CAFELTP experience indicates that considerable progress in building applied epidemiology program from a regional perspective can be achieved. The program is building international collaborations and training future public-health leaders.
